# Muscle Recruitment and Coordination following Constraint-Induced Movement Therapy with Electrical Stimulation on Children with Hemiplegic Cerebral Palsy: A Randomized Controlled Trial

**DOI:** 10.1371/journal.pone.0138608

**Published:** 2015-10-09

**Authors:** Kaishou Xu, Lu He, Jianning Mai, Xiaohua Yan, Ying Chen

**Affiliations:** 1 Department of Rehabilitation, Guangzhou Women and Children’s Medical Center, Guangzhou Medical University, Guangzhou, 510120, China; 2 Department of Neurology, Guangzhou Women and Children’s Medical Center, Guangzhou Medical University, Guangzhou, 510120, China; MUMC+ Academic Medical Center Maastricht, NETHERLANDS

## Abstract

**Objective:**

To investigate changes of muscle recruitment and coordination following constraint-induced movement therapy, constraint-induced movement therapy plus electrical stimulation, and traditional occupational therapy in treating hand dysfunction.

**Methods:**

In a randomized, single-blind, controlled trial, children with hemiplegic cerebral palsy were randomly assigned to receive constraint-induced movement therapy (n = 22), constraint-induced movement therapy plus electrical stimulation (n = 23), or traditional occupational therapy (n = 23). Three groups received a 2-week hospital-based intervention and a 6-month home-based exercise program following hospital-based intervention. Constraint-induced movement therapy involved intensive functional training of the involved hand during which the uninvolved hand was constrained. Electrical stimulation was applied on wrist extensors of the involved hand. Traditional occupational therapy involved functional unimanual and bimanual training. All children underwent clinical assessments and surface electromyography (EMG) at baseline, 2 weeks, 3 and 6 months after treatment. Surface myoelectric signals were integrated EMG, root mean square and cocontraction ratio. Clinical measures were grip strength and upper extremity functional test.

**Results:**

Constraint-induced movement therapy plus electrical stimulation group showed both a greater rate of improvement in integrated EMG of the involved wrist extensors and cocontraction ratio compared to the other two groups at 3 and 6 months, as well as improving in root mean square of the involved wrist extensors than traditional occupational therapy group (*p*<0.05). Positive correlations were found between both upper extremity functional test scores and integrated EMG of the involved wrist as well as grip strength and integrated EMG of the involved wrist extensors (*p*<0.05).

**Conclusions:**

Constraint-induced movement therapy plus electrical stimulation is likely to produce the best outcome in improving muscle recruitment and coordination in children with hemiplegic cerebral palsy compared to constraint-induced movement therapy alone or traditional occupational therapy.

**Trial registration:**

chictr.org ChiCTR-TRC-13004041

## Introduction

Cerebral palsy (CP) refers to a group of permanent disorders that affect the development of movement and posture that occur in the developing fetal or infant brain, leading to activity limitation[1]. Upper limb dysfunction is a common symptom in children with CP, especially in children with hemiplegic CP[2]. In addition, the uninvolved upper limb is usually used in the daily life, leading to the developmental disuse and disregard of the involved extremity, exacerbating the impaired hand dysfunction[3,4]. Due to sensory disturbance, hypertonia and muscle weakness of the involved upper limb, children with hemiplegic CP are impaired in the basic hand function such as grasping, reaching and manipulating[5]. Therefore, the hand dysfunction restricts their activities, participation, and quality of life.

In order to improve the hand function of children with hemiplegic CP, multiple therapeutic strategies with different outcomes have been developed[6–9]. One of them is constraint-induced movement therapy (CIMT) that was developed, proved to be an effective method, to treat children with hemiplegic CP[6–9]. The main principle of CIMT includes constraint of the uninvolved hand and intensive practice with the involved hand during a specified time period. In addition, traditional occupational therapeutic strategies are often applied to improving the hand function in children with hemplegic CP. Some evidences have demonstrated that CIMT and goal-directed occupational therapy (OT) home programs seem to be superior over standard care in improving upper limb and individualized outcomes[7].

Muscle weakness in children with CP is well documented[5]. Muscle strengthening in children with CP poses a challenge to clinical professionals due to the lack of muscle selectivity necessary for a specific strengthening program. Electrical stimulation has been proposed as a potentially useful modality for the improvement of muscle strength, sensory input, and muscle activation in patients with CP and stroke[10–15]. Therefore, CIMT in combination with electrical stimulation may be an effective alternative to CIMT alone.

Children with CP are characterized by insufficient muscle recruitment and activation consistent with low levels of surface myoelectric signals[16,17]. Braendvik et al[18] also reported that in children with CP, the involved arm had a low level of electromyography (EMG) amplitude and a weak muscle force during the maximum isometric voluntary contraction (MIVC). Surface EMG is a one-dimensional time series signal of biological electrical activity of the neuromuscular system recorded from the muscle surface. A previous study in which surface EMG was applied indicated that CIMT was effective in increasing the muscle activation in children with CP[19]. However, the efficacy of CIMT on muscle recruitment and activation remains unclear.

Previously, we compared CIMT plus electrical stimulation with CIMT and traditional OT, and examined the efficacy of them in improving hand performance in children with hemiplegic CP over different time frames[15]. Active ROM, grip strength[20], nine-peg hole test[21], upper extremity functional test[22], Peabody developmental motor scales[23], global rating scale[24], and social life ability scale[25] were selected as the outcome measures of the involved hand function, bimanual hand performance, and perceived changes by caregivers across a wide range of ages. While there is evidence to support the use of CIMT, CIMT with electrical stimulation and traditional OT to improve the hand function skills of children with hemiplegic CP, this study seeks to further investigate the underlying mechanism by which changes are made in muscle recruitment and activation.

Therefore, the objective of this study was to investigate the efficacy of muscle recruitment and coordination following CIMT, CIMT plus electrical stimulation and traditional OT in treating hand dysfunction in children with hemiplegic CP using surface EMG, and based on the above investigation, to analyze the relationship between hand function and surface myoelectric signals.

## Methods

### Participants

Children with CP aged 2 to 14 years from the neurology and rehabilitation department of a tertiary medical center were recruited for this study over a 1-year period. The inclusion criteria included a diagnosis of hemiplegic CP, the ability to extend the wrist ≥20° and the metacarpophalangeal joint 10° from full flexion, a 20%–80% functional difference between the involved and noninvolved hand, and to comply with study instructions. The exclusion criteria included uncontrolled seizures, severe health problems not typically associated with CP, contractures that limited functional arm and hand use, botulinum toxin injection in the upper limb during the last 6 months or who wished to receive it within the period of study, orthopedic surgery on their involved upper limb, visual and balance problems that would prevent them from carrying out the intervention or assessment. The study was approved by the medical ethical committee of Guangzhou Women and Children’s Medical Center and registered with www.chictr.org (trial registration number ChiCTR-TRC-13004041). The written informed consent was obtained from caregivers of all participants.

### Design

**Fig 1:**
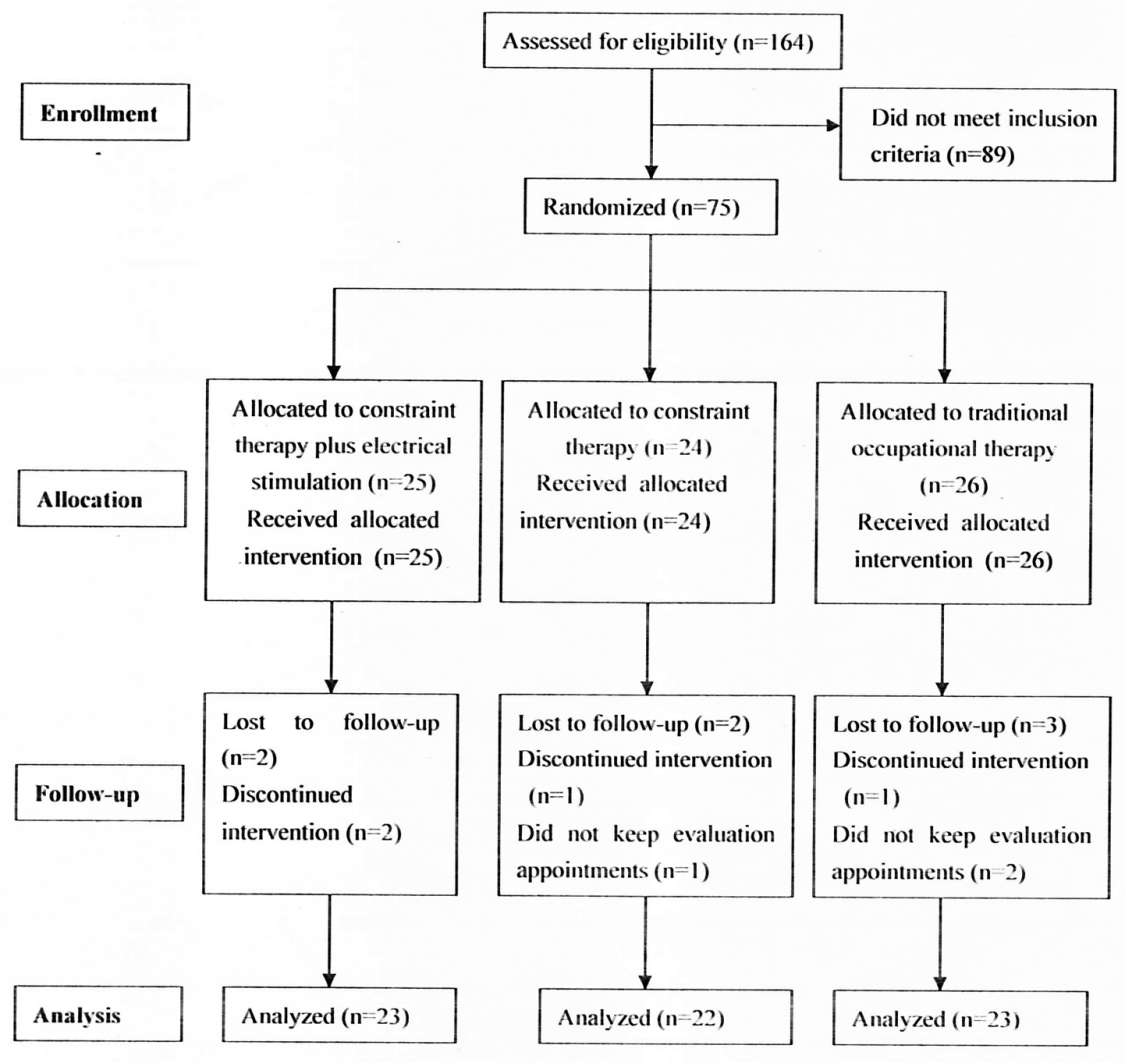
Intention-to-treat flowchart.

This study was a randomized, single-blind, controlled trial (Fig 1). The protocol for this trial and the CONSORT checklist are available as S1 CONSORT Checklist and S1 Protocol. Based on previous studies of using CIMT and OT on children with hemiplegic CP[6–9,15], we determined to have a power of 80% in statistical test and two-sided α = 0.05 to detect the difference in treatment effect. Assuming an estimated loss to follow-up of 20%, we calculated that we should enroll at least 75 children. All children who participated in the study were randomly divided into three groups: CIMT, CIMT plus electrical stimulation, or OT group. Among the 164 eligible candidates, a total of 68 children with hemiplegic CP (23 in the CIMT plus electrical stimulation group, 22 in the CIMT group, 23 in the OT group) were included in the final analysis. To minimize uneven distribution of known variables, the subjects were allocated in an unbiased manner a random number from computerized method of minimization[26]. The stratification included age (≤4 years and >4 years) and global rating scale scores[24] (≤5 and >5), a measure of involved hand severity created for the parent study of cerebral palsy outcomes. Scores range from 0 to 10.

All children underwent surface EMG analysis (MIVC of hand) and functional measures (clinical outcomes) in the neurology and rehabilitation department at baseline (about 2 weeks before the intervention). Outcome measures were administered by three independent occupational therapists who were not aware of the treatment group of each patient. All children underwent these assessments again at 2 weeks immediately after the hospital-based intervention, and at 3 and 6 months after the start of the intervention. After a 2-week therapist-based intervention at hospital, the intervention program was home-based. Demographic characteristics and functional performance were recorded, including age, gender, the difference between the involved and noninvolved hand on the global rating scale scores, the gross motor function classification system (GMFCS) level[1], and the manual ability classification system (MACS) level[9].

### Interventions

CIMT with orthosis[15] of the uninvolved hand or traditional OT was provided 3 hours a session, 5 days a week for 2 weeks at our hospital. In addition, at the end of the daily therapy, children were dismissed to a 1-hour home-based exercise program, which was extended to 2 hours a day for 6 months following hospital-based intervention. Parents were to complete the activity logs to monitor compliance. During CIMT, every child received personal instruction from professionals involving the specific practice of designated target movements. Children were engaged in therapeutic functional activities that provided the structured and intensive practice using the involved hand. The difficulty of the activity was increased by changing either temporal or spatial/accuracy task constraints when the target movement was performed successfully. Traditional OT program involved functional unimanual and bimanual training, and consisted of advice and treatment aimed at reducing spasticity, improving hand function and activities of daily life, and the provision of appropriate orthotics. Electrical stimulation was applied for 20 minutes a day, 5 times a week for 2 weeks, on extensors carpi radialis and extensors digitorum of the involved upper limb through a MyoTrac Infiniti dual-channel neuromuscular electrical stimulation unit and reusable carbonized-rubber electrodes. Frequencies were set at 50Hz, pulse rate 30 pulses per second with 300*μ*s of amplitude, and the amplitude to a maximum of 100mA. ON time was set to 12 seconds with 1 second of rise and decay and an OFF time of 12 seconds. Amplitude was increased slowly to the child’s tolerance without causing discomfort, and adjusted to induce the muscle contraction for all children. Three certified occupational therapists provided treatments for all the children. They also worked with the caregivers by follow-up telephone calls once every two weeks to monitor whether the home-based exercise program was done daily.

### Outcome measures

#### Clinical outcomes

Clinical outcome measures were hand-grip strength measured with sphygmomanometry[20], upper extremity functional test[22], and global rating scale[24]. The Intraclass Correlation Coefficient was 0.995 for upper extremity functional test, 0.919 for hand-grip strength measured with sphygmomanometry[15].

#### Surface EMG

**Fig 2:**
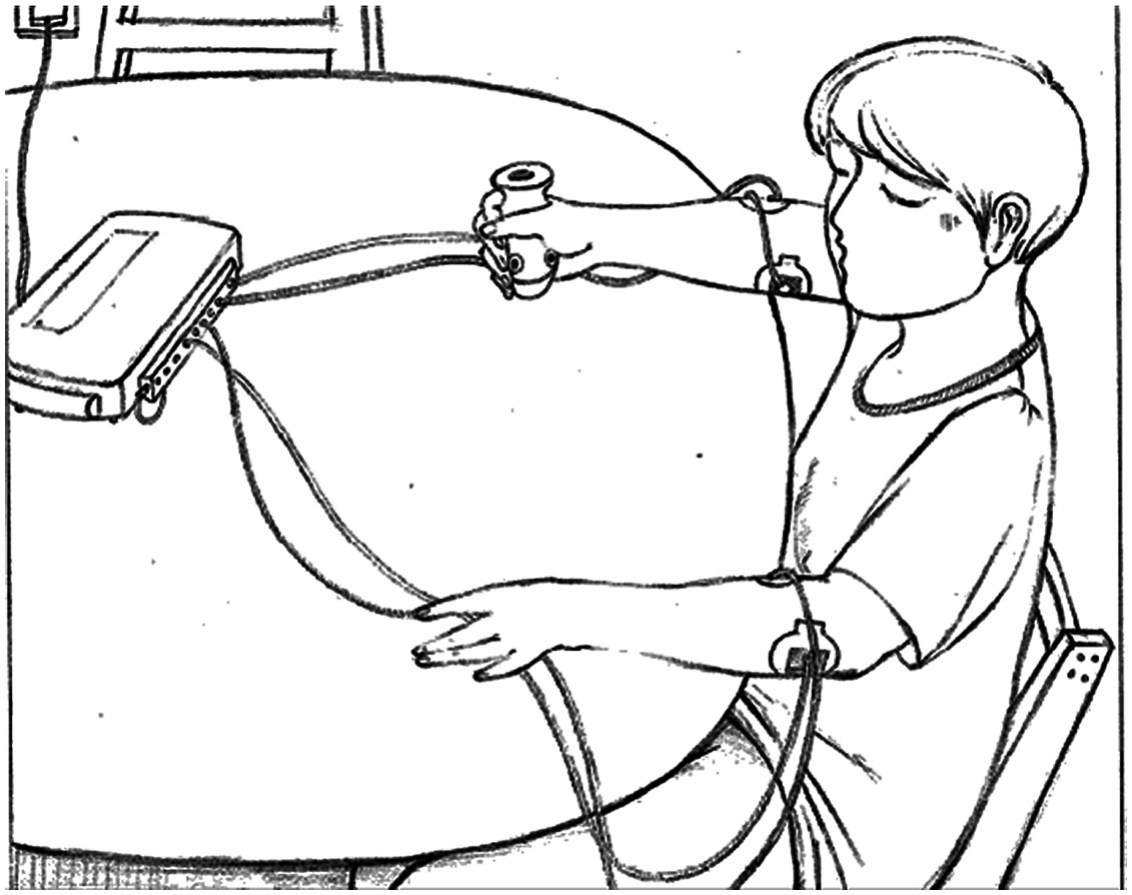
Test of surface electromyography.

The muscle recruitment and coordination was assessed with the surface EMG. The Flexcomp Infiniti surface EMG analysis system was used during the assessment phases. Surface electrodes were attached on the skin of wrist flexors and extensors. The uninvolved hand was measured first, followed by the involved one hand. The children were instructed to grip the cylindrically-shaped wood fish with maximum force to antagonize the examiner, aiming to produce MIVC of the wrist extensors and flexors (Fig 2). Each test lasted for 10 seconds followed by 10 seconds of rest, three times in a row.

Root mean square (RMS), Integrated EMG (iEMG), cocontraction ratio (CR) (CR = iEMG of wrist flexors / [iEMG of wrist extensors + iEMG of wrist flexors] ×100%) during MIVC were recorded and analyzed[27–32]. RMS approaches the quantification of the surface EMG signal by squaring the data, summing the squares, dividing the sum by the number of observations, and finally taking the square root[27–29]. The iEMG reflects the total discharge of motor units involved in movement and the discharge of each unit during a certain time[27–29]. CR could reflect the coordination and activation of agonist and antagonist muscle groups[30–32].

### Statistical analysis

Analysis was performed using IBM SPSS Statistics 20.0. If the values were normally distributed, One-Way ANOVA, repeated measure analysis of variances, analysis of covariance and post-hoc analysis (Bonferroni method) were used. Otherwise, nonparametric tests were used. Kruskal-Wallis test was applied to compare the differences of age and global rating scale scores. Chi-square test was used to compare the differences of gender, affected sides (right or left), GMFCS level, and MACS level among the three groups. One-Way ANOVA was applied to compare the differences of baseline data. Repeated measure analysis of variances was applied to compare the differences of measures before treatment, then at two weeks, three and six month intervals after treatment within each group. In order to exclude the effects caused by the different baseline data among the three groups, analysis of covariance was applied to compare the differences in measurements at two weeks, three and six months after treatment among the three groups (the baseline data being covariate). The correlation of surface EMG data and hand function was performed with Pearson correlation analyses. The P-values were 2-sided and considered statistically significant when less than 0.05 level.

## Results

### Demographics and baseline data

No significant differences (*p>0*.05, Table 1) were found in age, global rating scale scores, gender, affected sides (right or left), GMFCS level, and MACS level among the three groups obtained with Kruskal-Wallis test and Chi-square test, nor in hand-grip strength, upper extremity functional test scores, among the three groups obtained with One-Way ANOVA. Significant differences (*p*<0.05, Table 1) were found in iEMG of the involved wrist flexor and extensor during the MIVC at baseline among the three groups obtained with One-Way ANOVA. Yet no significant differences (*p>0*.05, Table 1) were found in other surface EMG data, such as RMS, CR, etc. during the MIVC at baseline among the three groups obtained with One-Way ANOVA.

**Table 1:** Demographic and baseline data: mean (SD) or n (%). MIVC, maximum isometric voluntary contraction; RMS, root mean square; iEMG, integrated electromyography.

Group	Constraint therapy plus electrical stimulation (n = 23)	Constraint therapy (n = 22)	Occupational therapy (n = 23)	P
Age (month)	56.8(34.0)	54.6(36.6)	54.7(30.8)	0.850
Male	7(30%)	7 (32%)	11 (48%)	0.467
Involved hand (right)	13(57%)	10(45%)	15 (63%)	0.409
Gross motor function classification system level I / level II	20(87%)/3(13%)	19(86%)/3(14%)	21(91%)/2(9%)	0.852
Manual ability classification system level I / level II / level III	3(13%)/17(74%)/3(13%)	4(18%)/16(73%)/2(9%)	3(13%)/16(70%)/4(17%)	0.125
Global rating scale (involved hand)	4.6(1.6)	3.8(1.8)	4.8(1.7)	0.123
Hand-grip strength (mmHg)	63.7(48.4)	65.5(56.0)	63.3(47.8)	0.988
Upper extremity functional test	35.0(22.9)	29.0(25.0)	37.8(30.5)	0.521
MIVC of involved hand				
RMS of involved wrist extensors (μV)	47.9(20.6)	47.0(16.8)	48.9(26.1)	0.958
RMS of involved wrist flexors (μV)	55.0(21.6)	54.3(17.1)	55.1(26.5)	0.992
RMS of uninvolved wrist extensors (μV)	20.7(9.3)	20.6(14.6)	25.8(21.8)	0.463
RMS of uninvolved wrist flexors (μV)	19.9(9.1)	21.9(15.2)	22.6(21.4)	0.836
iEMG of involved wrist extensors (μV·s)	911.8(386.4)	837.8(365.9)	614.3(401.0)	0.030
iEMG of involved wrist flexors (μV·s)	1045.0(451.9)	926.4(391.0)	729.4(441.6)	0.049
Cocontraction ratio	53.4(4.8)	52.7(2.2)	54.8(3.5)	0.157
iEMG of uninvolved wrist extensors (μV·s)	284.5(161.6)	258.2(182.3)	243.9(178.0)	0.726
iEMG of uninvolved wrist flexors (μV·s)	272.2(151.4)	250.0(185.9)	235.4(173.0)	0.762
MIVC of uninvolved hand				
RMS of involved wrist extensors (μV)	28.4(13.6)	30.6(17.5)	30.9(12.4)	0.815
RMS of involved wrist flexors (μV)	35.0(16.8)	32.7(15.6)	33.7(13.7)	0.880
RMS of uninvolved wrist extensors (μV)	77.5(19.2)	76.9(22.6)	77.9(24.1)	0.989
RMS of uninvolved wrist flexors (μV)	77.6(17.2)	77.9(22.8)	79.3(23.7)	0.961
iEMG of involved wrist extensors (μV·s)	595.3(254.1)	551.6(256.0)	570.2(201.5)	0.827
iEMG of involved wrist flexors (μV·s)	602.2(263.1)	572.9(255.5)	579.7(195.7)	0.911
Cocontraction ratio	47.9(2.6)	47.9(2.3)	48.1(1.7)	0.933
iEMG of uninvolved wrist extensors (μV·s)	1447.4(367.2)	1432.0(636.4)	1435.3(550.7)	0.995
iEMG of uninvolved wrist flexors (μV·s)	1319.9(275.1)	1328.8(588.3)	1339.3(537.7)	0.991

### Improvement of muscle recruitment and coordination

Compared with the results before the treatment during MIVC of the involved hand, RMS of both hands, iEMG and CR of the involved hand changed significantly after treatment for all the children in two weeks, three and six month respectively, as well as reducing in iEMG of the uninvolved hand was seen after six months of the treatment, obtained with repeated measure analysis of variances (*p*<0.05, Table 2).

**Table 2:** Change of root mean square (RMS, μV), integrated electromyography (iEMG, μV·s) and cocontraction ratio on maximum isometric voluntary contraction of involved hand at two weeks, three and six months: mean (SD).

Group	Constraint therapy plus electrical stimulation (n = 23)	Constraint therapy (n = 22)	Occupational therapy (n = 23)	P
RMS of involved wrist extensors				
Week 2-baseline	12.8(17.8)	9.1(9.7)	6.4(8.5)	0.246
Month 3-baseline	21.9(18.9)	16.8(11.3)	11.7(9.1)	0.049
Month 6-baseline	31.3(21.8)	24.9(14.6)	17.0(9.0)	0.015
RMS of involved wrist flexors				
Week 2-baseline	6.7(13.8)	6.6(8.0)	6.8±8.0	0.997
Month 3-baseline	17.3(17.2)	15.1(9.4)	14.7(10.3)	0.758
Month 6-baseline	27.1(25.0)	24.2(14.3)	23.2(11.8)	0.751
RMS of uninvolved wrist extensors				
Week 2-baseline	-4.0(9.0)	-4.0(4.0)	-3.2(2.6)	0.766
Month 3-baseline	-5.0(9.5)	-4.4(4.0)	-4.7(2.5)	0.881
Month 6-baseline	-8.8(8.6)	-6.5(5.3)	-7.0(4.2)	0.141
RMS of uninvolved wrist flexors				
Week 2-baseline	-3.8(7.8)	-3.9(4.9)	-3.7(2.9)	0.937
Month 3-baseline	-5.6(8.8)	-4.6(4.6)	-4.7(3.7)	0.611
Month 6-baseline	-8.4(9.5)	-6.9(5.8)	-7.0(5.7)	0.263
iEMG of involved wrist extensors				
Week 2-baseline	282.7(335.3)	159.9(180.7)	136.6(74.1)	0.087
Month 3-baseline	444.7(392.6)	244.4(199.9)	186.7(52.6)	0.007
Month 6-baseline	636.1(416.1)	321.9(256.1)	246.2(68.9)	0.000
iEMG of involved wrist flexors				
Week 2-baseline	200.6(254.1)	155.0(187.1)	135.0(81.3)	0.501
Month 3-baseline	308.5(321.7)	232.7(211.2)	185.8(82.6)	0.236
Month 6-baseline	428.4(360.1)	301.7(263.9)	242.8(77.1)	0.096
Cocontraction ratio				
Week 2-baseline	-2.7(4.2)	-0.6(1.2)	-1.2(2.5)	0.099
Month 3-baseline	-3.7(4.6)	-0.9(1.2)	-1.5(2.9)	0.000
Month 6-baseline	-5.0(5.4)	-1.2(1.3)	-1.7(2.7)	0.000
iEMG of uninvolved wrist extensors				
Week 2-baseline	3.7(62.2)	5.2(28.9)	5.0(25.1)	0.997
Month 3-baseline	-28.3(92.5)	-25.4(42.9)	-25.5(50.4)	0.985
Month 6-baseline	-59.3(92.3)	-54.4(56.5)	-52.9(51.9)	0.999
iEMG of uninvolved wrist flexors				
Week 2-baseline	4.6(36.4)	3.2(26.5)	3.9(30.9)	0.943
Month 3-baseline	-27.3(77.4)	-24.7(43.0)	-24.5(49.7)	0.974
Month 6-baseline	-53.3(106.0)	-55.2(52.6)	-54.4(44.2)	0.914

**Fig 3:**
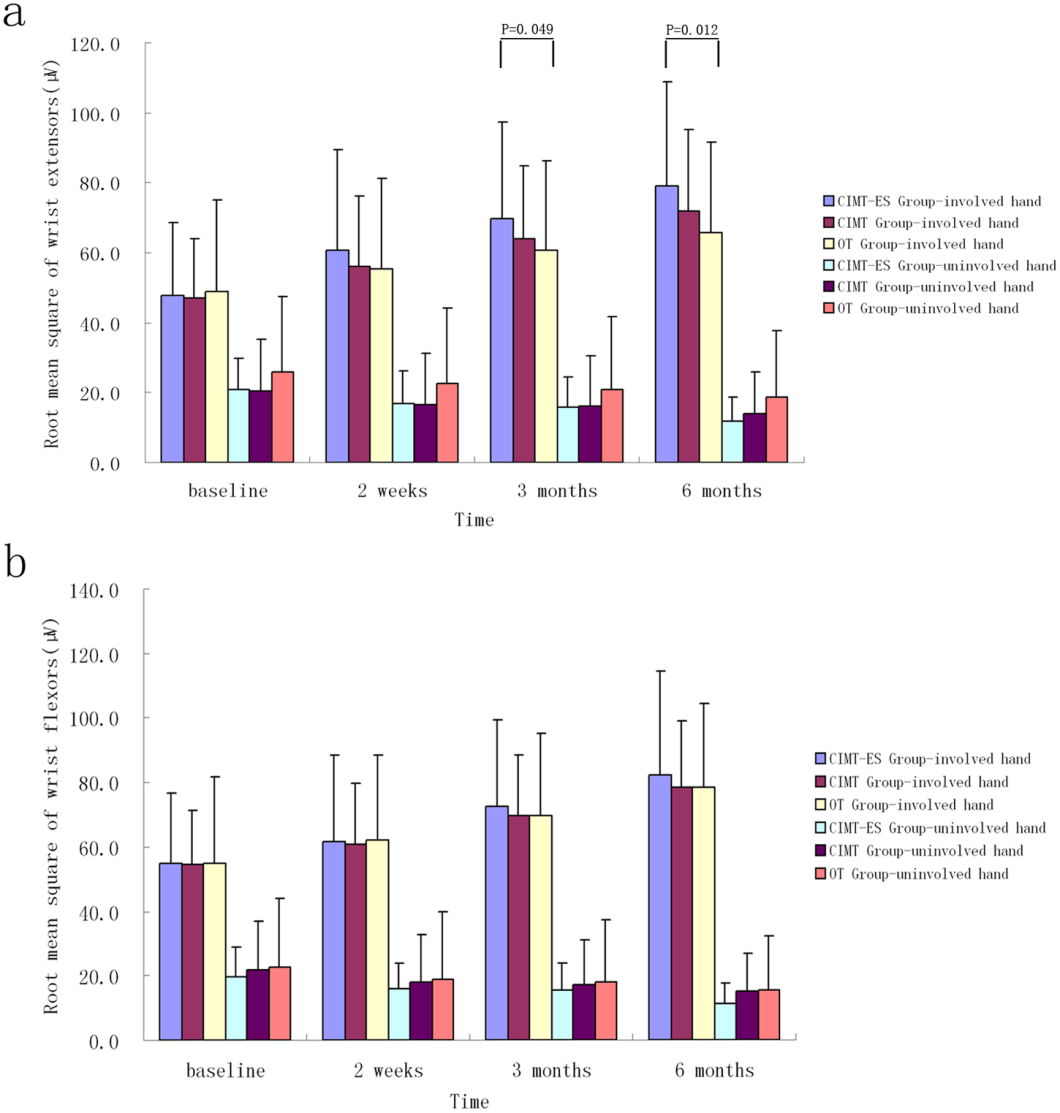
Changes in root mean square measured in the subjects' wrist extensors (a) and flexors (b) on maximum isometric voluntary contraction of the involved hand (mean ± SD). CIMT-ES group, constraint-induced movement therapy plus electrical stimulation group; CIMT group, constraint-induced movement therapy group; OT group, occupational therapy group.

**Fig 4:**
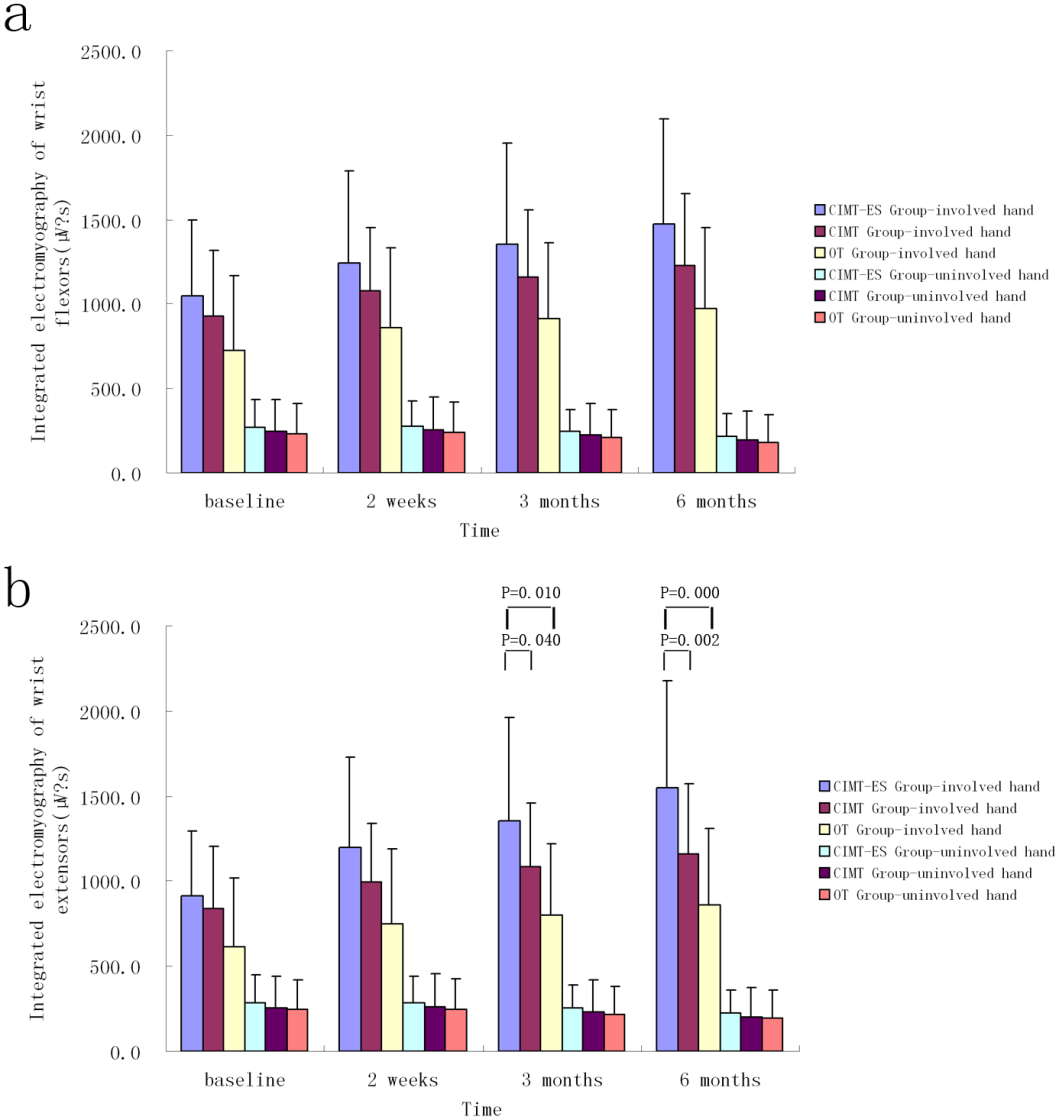
Changes in integrated electromyography measured in the subjects' wrist flexors (a) and extensors (b) on maximum isometric voluntary contraction of the involved hand (mean ± SD). CIMT-ES group, constraint-induced movement therapy plus electrical stimulation group; CIMT group, constraint-induced movement therapy group; OT group, occupational therapy group.

**Fig 5:**
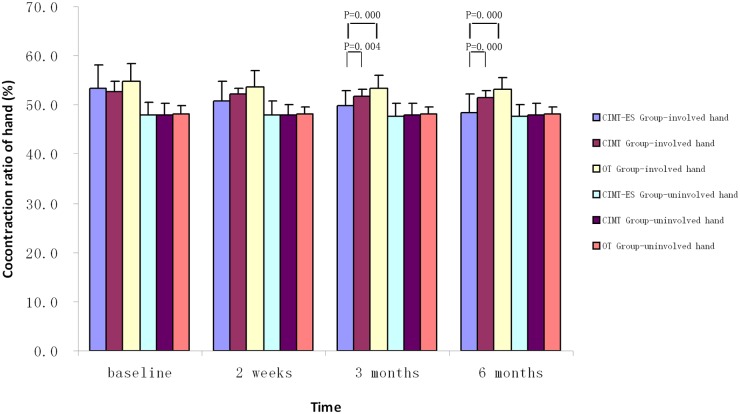
Changes in cocontraction ratio measured in the subjects' wrist on maximum isometric voluntary contraction of the involved hand and uninvolved hand (mean ± SD). CIMT-ES group, constraint-induced movement therapy plus electrical stimulation group; CIMT group, constraint-induced movement therapy group; OT group, occupational therapy group.

No significant differences were found in RMS and iEMG of the two wrists’ flexors and the uninvolved wrist extensors in each follow-up session among the three groups during MIVC of the involved hand, obtained with analysis of covariance (*p>0*.05, Table 2, Figs 3 and 4). However, the CIMT plus electrical stimulation group showed both a greater rate of improvement in the iEMG of the involved wrist extensors and CR compared to the OT group and CIMT group at three and six months, as well as improving in RMS of the involved wrist extensors than OT group, obtained with analysis of covariance (*p*<0.05, Figs 3–5).

In comparison with the results before the treatment during MIVC of the uninvolved hand, no significant changes were found in iEMG, RMS and CR measured in intervals of two weeks, three and six month after the treatment for all children obtained with repeated measure analysis of variances (*p>0*.05, Table 3). No significant differences were found in CR, RMS, and iEMG at each follow-up session among the three groups during MIVC of the uninvolved hand, obtained by the covariance analysis (*p>0*.05, Table 3).

**Table 3:** Change of root mean square (RMS, μV), integrated electromyography (iEMG, μV·s) and cocontraction ratio on maximum isometric voluntary contraction of uninvolved hand at two weeks, three and six months: mean (SD).

Group	Constraint therapy plus electrical stimulation (n = 23)	Constraint therapy (n = 22)	Occupational therapy (n = 23)	P
RMS of involved wrist extensors				
Week 2-baseline	0.6(14.6)	-0.6(5.4)	-0.4(4.8)	0.971
Month 3-baseline	-4.3(13.8)	-1.9(6.6)	-2.0(5.7)	0.331
Month 6-baseline	-6.8(11.6)	-4.7(6.3)	-4.1(6.0)	0.156
RMS of involved wrist flexors				
Week 2-baseline	1.9(13.7)	0.5(6.6)	-1.3(3.9)	0.406
Month 3-baseline	-3.2(13.3)	-1.5(7.0)	-1.9(6.3)	0.891
Month 6-baseline	-7.7(11.4)	-5.0(5.5)	-4.5(5.4)	0.338
RMS of uninvolved wrist extensors				
Week 2-baseline	0.2(4.5)	0.3(4.1)	0.2(4.6)	0.999
Month 3-baseline	2.2(6.0)	2.3(5.4)	2.3(5.5)	0.999
Month 6-baseline	2.9(4.4)	3.1(6.7)	2.8(6.3)	0.986
RMS of uninvolved wrist flexors				
Week 2-baseline	0.1(4.3)	0.2(2.0)	0.2(5.0)	0.992
Month 3-baseline	2.2(6.3)	2.2(7.2)	1.8(6.7)	0.974
Month 6-baseline	3.5(9.3)	3.5(8.3)	1.8(7.4)	0.752
iEMG of involved wrist extensors				
Week 2-baseline	-7.7(113.0)	-8.2(43.4)	-2.6(19.9)	0.953
Month 3-baseline	-35.6(180.9)	-17.7(79.0)	-7.3(42.9)	0.764
Month 6-baseline	-88.9(191.3)	-26.7(103.3)	-15.1(47.7)	0.128
iEMG of involved wrist flexors				
Week 2-baseline	-2.8(94.2)	-2.5(33.3)	-2.3(18.6)	1.000
Month 3-baseline	-15.3(139.3)	-3.8(50.4)	-10.7(40.1)	0.953
Month 6-baseline	-71.0(181.4)	-36.9(90.8)	-18.7(44.8)	0.332
Cocontraction ratio				
Week 2-baseline	0.0(1.6)	0.0(0.2)	0.0(0.2)	0.979
Month 3-baseline	-0.1(1.7)	0.0(0.9)	0.0(0.5)	0.942
Month 6-baseline	-0.2(1.7)	0.1(0.8)	0.0(0.5)	0.663
iEMG of uninvolved wrist extensors				
Week 2-baseline	2.9(95.3)	3.9(38.4)	2.2(45.8)	0.996
Month 3-baseline	3.7(147.1)	4.1(74.3)	4.5(56.8)	1.000
Month 6-baseline	7.9(159.8)	7.6(61.1)	8.0(64.8)	1.000
iEMG of uninvolved wrist flexors				
Week 2-baseline	1.1(80.7)	1.3(44.1)	1.2(44.1)	1.000
Month 3-baseline	1.9(98.9)	1.5(39.3)	1.8(59.7)	1.000
Month 6-baseline	7.2(108.4)	7.0(41.4)	6.9(62.4)	1.000

### Relationship between surface myoelectric signals and hand function

**Fig 6:**
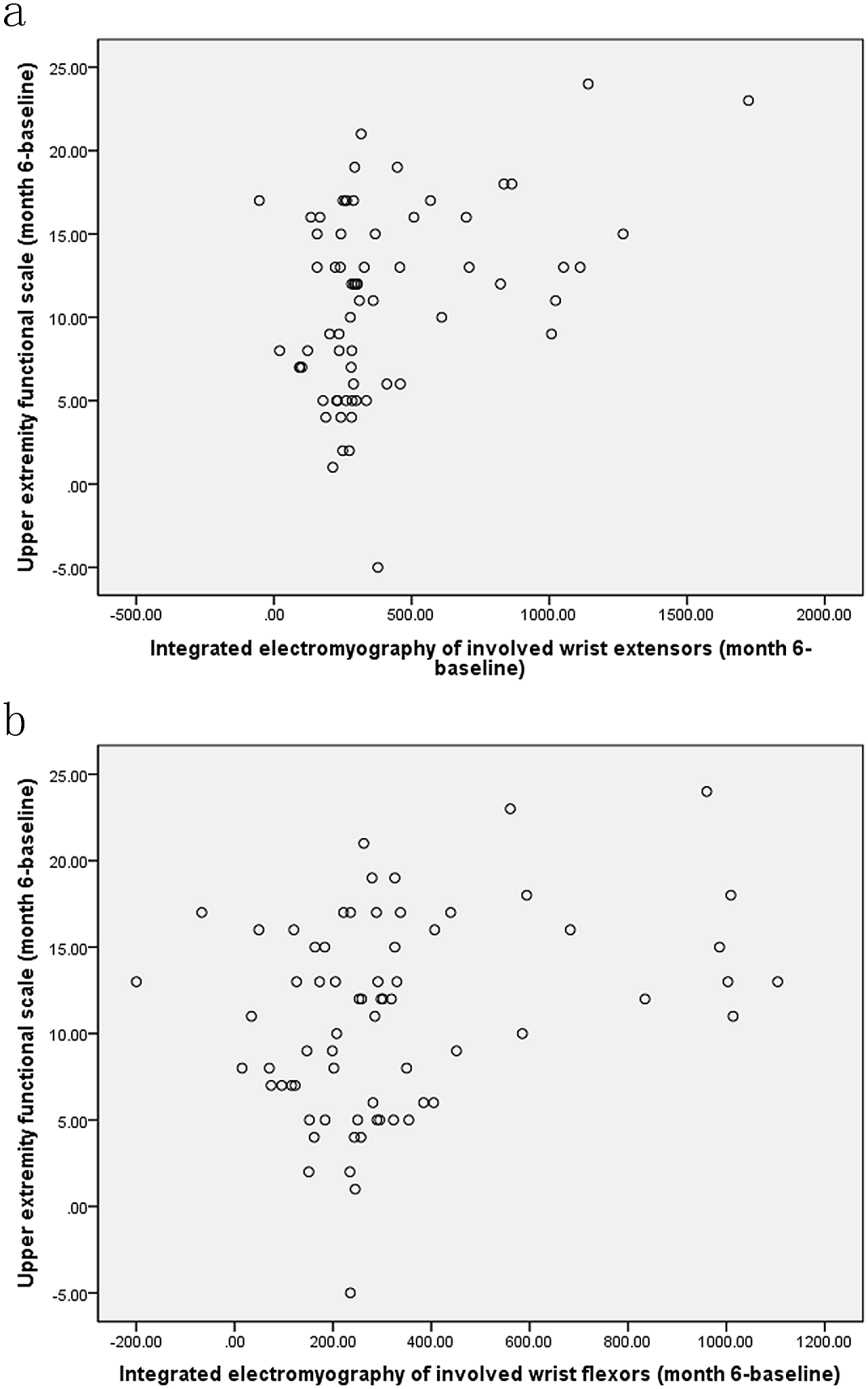
Simple scatter of changes between upper extremity functional scale scores and integrated electromyography (μV·s) of involved wrist extensors (a) and flexors (b) at six month on maximum isometric voluntary contraction of the involved hand.

**Fig 7:**
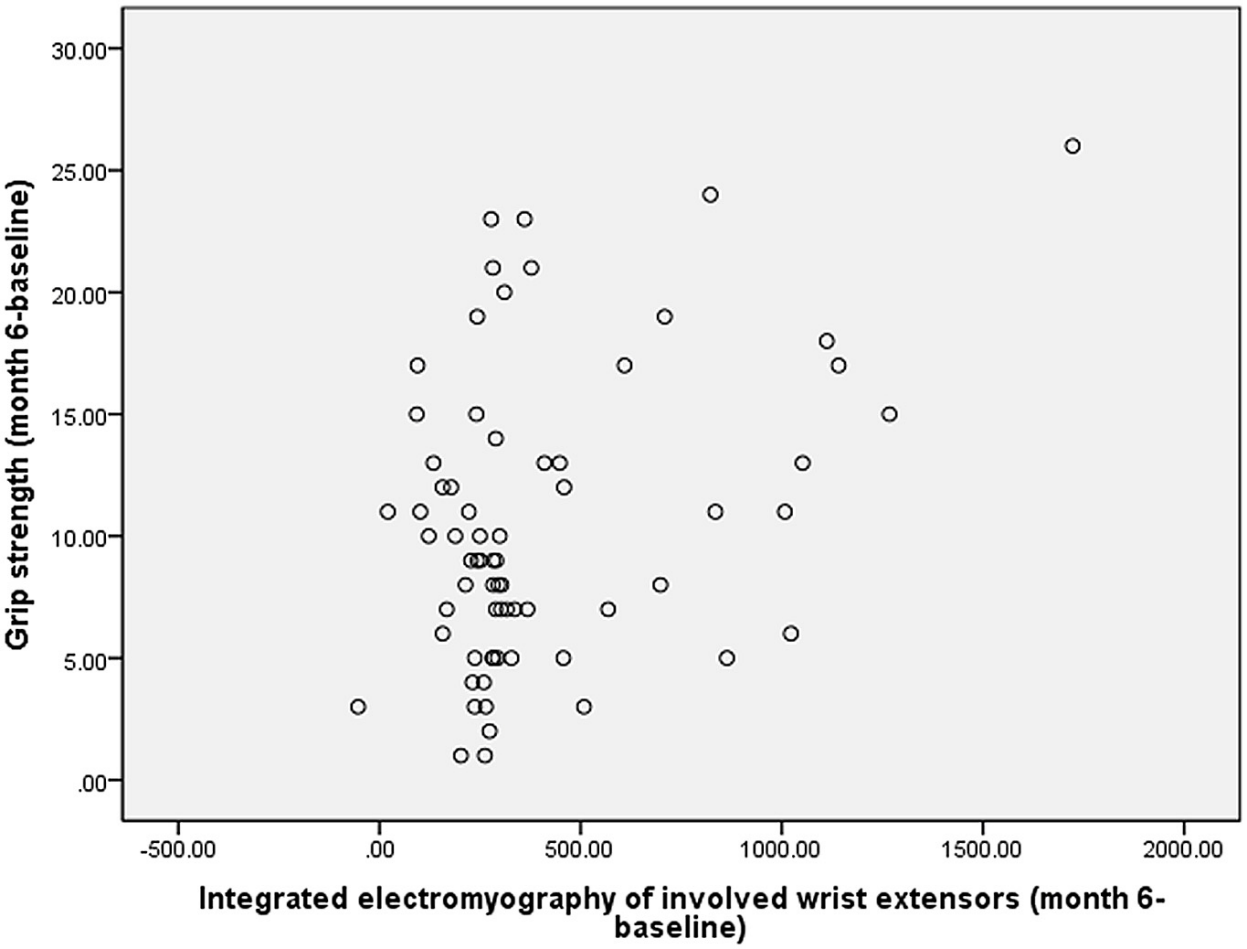
Simple scatter of changes between grip strength (in mmHg) and integrated electromyography (μV·s) of involved wrist extensors at six month on maximum isometric voluntary contraction of the involved hand.

The mean improvements between baseline and end of follow-up were respectively 10.5 mmHg for grip strength, 11.0 for upper extremity functional test scores, 402.6 and 324.7 μV·s for iEMG of involved wrist extensors and flexors during MIVC of the involved hand in all the children. During MIVC of the involved hand, the improvement shown in the scores of upper extremity functional test was positively correlated with the increase of iEMG in the involved wrist extensors (*r = 0*.301, *p = 0*.013, Fig 6) and flexors (*r = 0*.395, *p = 0*.001, Fig 6) after six months of treatment for all the children. The improvement of grip strength and the increase of iEMG of the involved wrist extensors (*r = 0*.362, *p = 0*.002, Fig 7), obtained with Pearson analysis, was also positively correlated.

## Discussion

The results of this study demonstrated that the use of CIMT plus electrical stimulation, CIMT and traditional OT could strengthen muscle recruitment and coordination of the involved hand, and the bimanual isolated movement control. Pearson's analysis indicated that the global functional improvement of the involved arm and hand was associated strongly with the increase of muscle recruitment. The results also suggest that surface EMG is an effective method in evaluating hand function in children with hemiplegic CP. The CIMT plus electrical stimulation showed a superiority over the CIMT alone and traditional OT, though it might be slightly more expensive and take longer time.

Analysis of covariance was applied to compare the differences of EMG data after treatment among the three groups, excluding the effects caused by the different baseline data among the three groups. Therefore, any significant change rate among the three groups should be attributed to the different intervention.

Reflecting the surface myoelectric signals changes in time dimension, the RMS is positively correlated with the proportion of type II fibers[27–29]. The results showed that RMS was elevated by all three treatments. Moreover, CIMT plus electrical stimulation showed an improvement in the RMS instead of only OT at 3 and 6 months of treatment. It indicates that CIMT plus electrical stimulation could effectively enhance the synchronization of motor unit recruitment and exciting rhythms. The results suggested that electrical stimulation should be probably an effective potential method to raise the shrunken type II fibers of children with CP.

The iEMG is a reflection of the discharge amount of motor unit participation in activities during a certain period in the muscle, depending on the change of EMG amplitude[27–29]. During MIVC of the involved hand, iEMG of the involved wrist flexors and extensors was obviously improved in all three groups after 2 weeks, 3 and 6 months of treatment. It indicates that CIMT plus electrical stimulation, CIMT and OT can quickly increase the discharge amount of the participating motor unit when the involved wrist flexors and extensors are shortened and remain so till to 6 months after treatment. When the involved hand grasping maximally 6 months after treatment, the iEMG of the uninvolved wrist flexors and extensors decreased significantly, in comparison with those at baseline, which suggested the voluntary movements control capacity of the uninvolved hand was also strengthened. Among the three treatment methods, CIMT plus electrical stimulation showed the greatest improvement in the iEMG of the involved wrist extensors. It was reported that iEMG was positively correlated with muscle strength when muscle shortening occured[32]. In our study, we also found that the increase of iEMG was positively correlated with the improvement of the grip strength of the involved hand after 6 months of treatment. The improvement of the involved hand function (upper extremity function test) also positively correlated with the increase of iEMG of the involved wrist flexors and extensors during MIVC. It indicates the increase of the motor unit recruitment number and discharge quantity in wrist flexors and extensors is directly related to the overall function of the upper limbs. The findings in our study are consistent with previous observations[19,32,33].

The surface EMG is considered to be an ideal and credible method for the evaluation of CR[30–33]. Excessive cocontraction could impact the performance of motor function. Elder et al[34] reported that the antagonist muscle had higher levels of co-activation in children with CP, suggesting that the agonist muscle was not fully activated. CIMT plus electrical stimulation, CIMT and OT could improve the coordination of the involved wrist in the short term, reduce the ratio of wrist flexors participated in cocontraction, and thereby improve the movement efficiency. Furthermore, CIMT plus electrical stimulation showed superiority in reducing the level of CR in the wrist flexors, maintaining the good position of the wrist muscle force production in children with CP.

Children with hemiplegic CP due to brain damage are usually affected by associated reaction or mirror movements (i.e., the unconscious and uncontrolled movement of one hand that follows the same pattern of the other hand). It impacts bimanual isolated movement control when the hands are required to do different movements (e.g., one hand stabilizing a piece of paper while the other writing)[35]. As represented in Figs 3 and 4, RMS and iEMG of the involved wrist extensors and flexors increased after the treatment whereas those of the uninvolved wrist extensors and flexors decreased. It indicates that bimanual isolated movement control ability might be improved.

Electrical stimulation induced the wrist extensors contraction of repetition in this study, which would input the sensory information to the brain by the mechanical stimulation. Evidence from this study indicates that electrical stimulation may lead to better response to CIMT. One of the mechanisms may be proposed by the increase of sensory input[10,36–38]. The evidence indicates that electrical stimulation can excite large sensory fibers, predominantly in the A-beta range[10,36]. Through the cutaneous stimulation of muscles, it increases the excitability of the sensorimotor cortex[37]. Dobkin[38] suggested that the patterned sensory inputs of electrical stimulation should be beneficial to synaptic and biological adaptations within the cortex. Khaslavskaia et al[37] also reported that repeated electrical stimulation could significantly increase motor evoked potentials of the muscle. Another possible mechanism may be that CIMT with electrical stimulation results in a more mature muscle activation pattern by increasing motor unit recruitment and synchronization, selective recruitment of type II fibers (fast twitch, large diameter fibers), and facilitates structural reorganization of brain networks[17,32,34,39–43]. Rose et al[17] found that the muscle strength in children with CP was weakened, suggesting ineffective and incomplete activation. Sutcliffe et al[43] showed that a shift to or persistence of contralateral cortical activity for involved hand movement was important for CIMT mechanism of action. We thus purported that our subjects could gain the most benefits when they practiced CIMT simultaneously with electrical stimulation.

Most of surface myoelectric signals in the uninvolved wrist did not show statistical significant differences in the 3 groups during MIVC, though it seems to indicate that trend. This may be explained by the “ceiling effect” of muscle excitement and “floor effect” of muscle silence in the uninvolved wrist.

### Study limitations

The limitations of this study are as follows: (a) as to the group setting, the present study did not include a single electrical stimulation group since electrical stimulation was not yet well developed in children with CP; (b) this study only focused on the involved hand function. Therefore, it would be much useful to investigate the bimanual hands function using the grasping and visual-motor integration subtests of the Peabody motor developmental scales in the future. (c) some children had to be engaged in interesting activities to distract them from a degree of fear of electrical stimulation; (d) the potential confounding comorbidity of the participants was not evaluated; (e) the sample size of each group was relatively small. So, it would be high-powered to investigate a greater number of children. Further research is necessary.

## Conclusions

CMIT plus electrical stimulation, CIMT and traditional OT all improve the muscle recruitment and coordination of involved hand and the bimanual isolated movement control ability in children with hemiplegic CP. However, CIMT plus electrical stimulation is the most effective. Muscle recruitment is closely related to the global functional improvement of the involved hand.

## Supporting Information

S1 CONSORT Checklist (DOC)

S1 Protocol Trial Protocol in Chinese. (DOC)

S2 Protocol English translation of the original protocol. (DOC)
