# Supplementary material for: Muscle Recruitment and Coordination following Constraint-Induced Movement Therapy with Electrical Stimulation on Children with Hemiplegic Cerebral Palsy: A Randomized Controlled Trial
Source: PLoS One. 2015 Oct 9;10(10):e0138608. doi: 10.1371/journal.pone.0138608 (PMC4599892; doi:10.1371/journal.pone.0138608)
Supplement: S1 Protocol — (DOC) [file pone.0138608.s002.doc]

**限制-诱导运动疗法结合电刺激对偏瘫型脑瘫患儿上肢肌肉募集和协调功能影响的随机对照研究**

**研究背景**

脑性瘫痪(简称脑瘫)是指一组发生于发育中胎儿或婴儿脑的非进行性功能紊乱致使引起活动受限的运动和姿势发育性永久障碍。上肢功能障碍在脑性瘫痪患儿中很常见，尤其是偏瘫患儿。脑瘫患儿的特点是低水平的表面肌电信号并伴有不充分的肌肉募集和激活。之前应用表面肌电图的研究表明限制-诱导运动疗法可有效提高脑瘫患儿的肌肉激活水平。然而限制-诱导疗法对于肌肉募集和激活的效能仍然不清楚。虽然有证据支持限制-诱导疗法、限制诱导疗法结合电刺激、作业治疗的应用可改善偏瘫患儿手功能，但本研究旨在进一步调查由肌肉募集和激活造成手功能变化的潜在机制。本研究结果有助于临床人员解释应用限制-诱导疗法、限制诱导疗法结合电刺激、作业治疗后功能改善潜在的肌肉募集的神经机制变化。

**研究目的**

我们通过表面肌电图 监测和分析偏瘫型脑性瘫痪患儿接受限制-诱导疗法、限制-诱导疗法结合电刺激、作业治疗三种技术治疗前后的上肢肌肉收缩时的募集和肌群协调功能状况，以及分析手功能和表面肌电信号的关系。

**假设**

在前期研究基础上，我们假设偏瘫患儿在医院进行限制-诱导疗法结合电刺激治疗2周以及6个月的家庭训练与仅进行限制-诱导疗法或传统作业治疗法相比较，将可明显增加肌肉募集和激活。

**研究终点**

**主要结局测评：**表面肌电图分析（手的最大等长收缩）和功能测评（血压计测试法的抓握力量、上肢功能测试、整体评价量表）。时间点分别为：治疗前2周、医院治疗后2周、3个月和6个月。

**次要结局测评：**副作用。

**研究设计**

本研究为前瞻性的随机单盲对照研究。所有儿童由计算机随机生成数字随机分为三组：CIMT、CIMT-ES、OT。

**地点：**中国广州市妇女儿童医疗中心神经康复科门诊。

**研究人数**

75例患儿

**入选标准**

确诊为偏瘫型脑瘫；从完全屈曲位，患手腕关节背伸≥20°，掌指关节伸展≥10°；患手与健手功能的整体评价量表得分之间的差异为20% - 80%之间；及患儿家长能够签署研究同意书和配合研究。

**排除标准**

未控制的癫痫发作；患有与脑性瘫痪不相关的其它严重的健康问题；影响患手功能性使用的固定挛缩；在最近6个月接受过肉毒毒素注射，或在研究期间内准备接受A型肉毒毒素注射；患侧上肢接受过骨科手术；影响康复干预和评定的视觉和平衡障碍。

**技术路线**

随机分组

偏瘫型脑瘫患儿

符合入选标准

入选(n=75)

计算机分层

CIMT组

(n=25)

OT组

(n=25)

CIMT-ES组

(n=25)

数据分析

数据整理、统计、撰写论文

排除

年龄

手功能

No

Yes

康复评定与治疗

**干预**

患儿在我们医院应用CIMT限制健手或传统OT 3 小时每次，每周5次，连续2周。另外，在每天治疗结束后，患儿回家进行1小时家庭康复训练并在医院干预2周后，延长至每天2小时，连续6个月，家长定期填写活动日志表以监测训练。在CIMT期间，每个儿童从专业人员里接受个体化指导，包括指定目标运动的具体实践。儿童从事功能性活动，这些活动可为患侧手提供结构化和高强度的实践训练。当目标运动已经成功完成时，就通过改变任务的时间、或空间和精确度的任务限制以提高活动的难度。传统的OT项目包括单手及双手训练，和旨在减少痉挛的建议和治疗，改善手功能及日常生活的活动，以及合适矫形器的提供。采用MyoTrac Infiniti双通道神经肌肉电刺激装置和可重复使用的碳化橡胶电极对患侧手的桡侧腕伸肌和指伸肌进行电刺激，每天为20min，每周5次，连续2周。为所有儿童的治疗由三个已被认证的作业治疗师提供，同时作业治疗师每两周通过电话随访以监测日常家庭训练计划是否完成。

**研究风险**

目前为止，已知CIMT、CIMT-ES及OT没有风险或副作用。

**统计分析：样本量和检验效能**

检测患手协同收缩率在基线期和治疗6个月后之间的差异（在前期研究基础上）。

双侧检验为5%（α=0.05）效能为80%（β=0.20）。60个患儿（例：CIMT-ES组，20例；CIMT组，20例；OT组，20例）必须全部包括。考虑到25%的脱落率，共75例。

**签名页**

**研究人员**

**徐开寿**

广州医科大学，广州市妇女儿童医疗中心康复科（510120）

**何璐**

广州医科大学，广州市妇女儿童医疗中心康复科（510120）

**麦坚凝**

广州医科大学，广州市妇女儿童医疗中心神经科（510120）

**严晓华**

广州医科大学，广州市妇女儿童医疗中心康复科（510120）

**陈莹**

广州医科大学，广州市妇女儿童医疗中心康复科（510120）

**广州 2013年9月14日**
