# Supplementary material for: Muscle Recruitment and Coordination following Constraint-Induced Movement Therapy with Electrical Stimulation on Children with Hemiplegic Cerebral Palsy: A Randomized Controlled Trial
Source: PLoS One. 2015 Oct 9;10(10):e0138608. doi: 10.1371/journal.pone.0138608 (PMC4599892; doi:10.1371/journal.pone.0138608)
Supplement: S2 Protocol — (DOC) [file pone.0138608.s003.doc]

**Muscle recruitment and coordination following constraint-induced movement therapy with electrical stimulation on children with hemiplegic cerebral palsy: a randomized controlled trial**

**Summary**

**Background**

Cerebral palsy (CP) refers to a group of permanent disorders that affect the development of movement and posture that occur in the developing fetal or infant brain, leading to activity limitation. Upper limb dysfunction is a common symptom in children with CP, especially in children with hemiplegic CP. Children with CP are characterized by insufficient muscle recruitment and activation consistent with low levels of surface myoelectric signals. A previous study in which surface electromyography (EMG) was applied indicated that constraint-induced movement therapy (CIMT) was effective in increasing the muscle activation in children with CP. However, the efficacy of CIMT on muscle recruitment and activation remains unclear. While there is evidence to support the use of CIMT, CIMT with electrical stimulation and traditional occupational therapy (OT) to improve the hand function skills of children with hemiplegic CP, this study seeks to further investigate the underlying mechanism by which changes are made in muscle recruitment and activation. The finding of this study will help clinicians elucidate the neuromechanism changes in muscle recruitment underlying functional improvement after CIMT, CIMT plus electrical stimulation and OT.

**Purpose**

we hypothesize that a 2-week hospital-based treatment course with CIMT plus electrical stimulation and a 6-month home-based exercise program will significantly improve muscle recruitment and activation in children with hemiplegic cerebral palsy compared to CIMT alone or traditional OT.

**Study end-points**

**Primary outcome measures:** surface EMG analysis (maximum isometric voluntary contraction of hand) and functional measures (hand-grip strength measured with sphygmomanometry, upper extremity functional test, global rating scale) (time frame: about 2 weeks before the intervention, 2 weeks immediately after the hospital-based intervention, and at 3 and 6 months after the start of the intervention).

**Secondary outcome measures:** Side effects.

**Study design**

Prospective, randomized, single-blind, controlled study. All children who participated in the study were randomly divided into three groups: CIMT, CIMT plus electrical stimulation, or OT group based on computer-generated random numbers.

Setting: Outpatient clinic, Department of Neurology and Rehabilitation, Guangzhou Women and Children’s Medical Center, Guangzhou.

**Study population**

Seventy-five patients.

**Inclusion criteria**

A diagnosis of hemiplegic CP, the ability to extend the wrist ≥20° and the metacarpophalangeal joint 10° from full flexion, a 20% - 80% functional difference between the involved and noninvolved hand, and written informed consent form could be got from parents and to comply with study instructions.

**Exclusion criteria**

Uncontrolled seizures, severe health problems not typically associated with CP, contractures that limited functional arm and hand use, botulinum toxin injection in the upper limb during the last 6 months or who wished to receive it within the period of study, orthopedic surgery on their involved upper limb, visual and balance problems that would prevent them from carrying out the intervention or assessment.

**Interventions**

CIMT with orthosis of the uninvolved hand or traditional OT was provided 3 hours a session, 5 days a week for 2 weeks at our hospital. In addition, at the end of the daily therapy, children were dismissed to a 1-hour home-based exercise program, which was extended to 2 hours a day for 6 months following hospital-based intervention. Parents were to complete the activity logs to monitor compliance. During CIMT, every child received personal instruction from professionals involving the specific practice of designated target movements. Traditional OT program involved functional unimanual and bimanual training. Electrical stimulation was applied for 20 minutes a day, 5 times a week for 2 weeks, on extensors carpi radialis and extensors digitorum of the involved upper limb through a MyoTrac Infiniti dual-channel neuromuscular electrical stimulation unit and reusable carbonized-rubber electrodes.

**Study flowchart**

Study candidate

Randomized (n=75)

Allocated to constraint therapy (n=25)

Allocated to constraint therapy plus electrical stimulation (n=25)

Allocated to traditional occupational therapy (n=25)

Received allocated

intervention (n=26)

Analyzed

Analyzed

Analyzed

**Signature page**

**Investigators**

**Kaishou Xu**,

Director, Department of Rehabilitation,

Guangzhou Women and Children’s Medical Center, Guangzhou Medical University.

**Lu He**,

Staff member, Department of Rehabilitation,

Guangzhou Women and Children’s Medical Center, Guangzhou Medical University.

**Jianning Mai**,

Director, Department of Neurology,

Guangzhou Women and Children’s Medical Center, Guangzhou Medical University.

**Xiaohua Yan,**

Staff member, Department of Rehabilitation,

Guangzhou Women and Children’s Medical Center, Guangzhou Medical University.

**Ying Chen,**

Staff member, Department of Rehabilitation,

Guangzhou Women and Children’s Medical Center, Guangzhou Medical University.

**Guangzhou 14.09.2013**

**Background**

Cerebral palsy (CP) refers to a group of permanent disorders that affect the development of movement and posture that occur in the developing fetal or infant brain, leading to activity limitation. Upper limb dysfunction is a common symptom in children with CP, especially in children with hemiplegic CP. Children with CP are characterized by insufficient muscle recruitment and activation consistent with low levels of surface myoelectric signals. A previous study in which surface electromyography (EMG) was applied indicated that constraint-induced movement therapy (CIMT) was effective in increasing the muscle activation in children with CP. However, the efficacy of CIMT on muscle recruitment and activation remains unclear. While there is evidence to support the use of CIMT, CIMT with electrical stimulation and traditional occupational therapy (OT) to improve the hand function skills of children with hemiplegic CP, this study seeks to further investigate the underlying mechanism by which changes are made in muscle recruitment and activation. The finding of this study will help clinicians elucidate the neuromechanism changes in muscle recruitment underlying functional improvement after CIMT, CIMT plus electrical stimulation and OT.

**Purpose**

We investigate the efficacy of muscle recruitment and coordination following CIMT, CIMT plus electrical stimulation and OT in treating hand dysfunction in children with hemiplegic CP using surface EMG, and based on the above investigation, to analyze the relationship between hand function and surface myoelectric signals.

**Hypothesis**

Based on our pilot study we assume, that a 2-week hospital-based treatment course with CIMT plus electrical stimulation and a 6-month home-based exercise program will significantly improve muscle recruitment and activation in children with hemiplegic cerebral palsy compared to CIMT alone or traditional OT.

**Study end-points**

**Primary outcome measures:** surface EMG analysis (maximum isometric voluntary contraction of hand) and functional measures (hand-grip strength measured with sphygmomanometry, upper extremity functional test, global rating scale) (time frame: about 2 weeks before the intervention, 2 weeks immediately after the hospital-based intervention, and at 3 and 6 months after the start of the intervention).

**Secondary outcome measures:** Side effects.

**Study design**

Prospective, randomized, single-blind, controlled study. All children who participated in the study were randomly divided into three groups: CIMT, CIMT plus electrical stimulation, or OT group based on computer-generated random numbers.

Setting: Outpatient clinic, Department of Neurology and Rehabilitation, Guangzhou Women and Children’s Medical Center, Guangzhou.

**Study population**

Seventy-five patients.

**Inclusion criteria**

A diagnosis of hemiplegic CP, the ability to extend the wrist ≥20° and the metacarpophalangeal joint 10° from full flexion, a 20% - 80% functional difference between the involved and noninvolved hand, and written informed consent form could be got from parents and to comply with study instructions.

**Exclusion criteria**

Uncontrolled seizures, severe health problems not typically associated with CP, contractures that limited functional arm and hand use, botulinum toxin injection in the upper limb during the last 6 months or who wished to receive it within the period of study, orthopedic surgery on their involved upper limb, visual and balance problems that would prevent them from carrying out the intervention or assessment.

**Study flowchart**

Assessed for eligibility

Did not meet inclusion criteria

Randomized (n=75)

Allocated to constraint therapy (n=25)

Allocated to constraint therapy plus electrical stimulation (n=25)

Allocated to traditional occupational therapy (n=25)

Received allocated

intervention (n=26)

Lost to follow-up

Lost to follow-up

Lost to follow-up

Analyzed

Analyzed

Analyzed

**Interventions**

CIMT with orthosis of the uninvolved hand or traditional OT was provided 3 hours a session, 5 days a week for 2 weeks at our hospital. In addition, at the end of the daily therapy, children were dismissed to a 1-hour home-based exercise program, which was extended to 2 hours a day for 6 months following hospital-based intervention. Parents were to complete the activity logs to monitor compliance. During CIMT, every child received personal instruction from professionals involving the specific practice of designated target movements. Traditional OT program involved functional unimanual and bimanual training. Electrical stimulation was applied for 20 minutes a day, 5 times a week for 2 weeks, on extensors carpi radialis and extensors digitorum of the involved upper limb through a MyoTrac Infiniti dual-channel neuromuscular electrical stimulation unit and reusable carbonized-rubber electrodes.

**Risks and inconveniences**

Up to now, there are no known risks or side effects by CIMT, CIMT plus electrical stimulation and OT.

**Statistical analyses: sample size and power calculation**

To detect a difference in the cocontraction ratio of involved hand between baseline and after 6 months of therapy (based on the data of the pilot study) at a two-sided significance level of 5% (α=0.05) with a power of 80% (β=0.20) a total of 60 patients has to be recruited (i.e. 20 patients in the CIMT plus electrical stimulation group, 20 patients in the CIMT group, 20 patients in the OT group, respectively). Considering a drop-out rate of 25%, a total of 75 patients should be included into the trial.
